# Synchronizing Two Superconducting Qubits through a Dissipating Resonator

**DOI:** 10.3390/e23080998

**Published:** 2021-07-31

**Authors:** Benedetto Militello, Anna Napoli

**Affiliations:** 1 Dipartimento di Fisica e Chimica—Emilio Segrè, Universitá degli Studi di Palermo, Via Archirafi 36, 90123 Palermo, Italy; anna.napoli@unipa.it; 2 INFN Sezione di Catania, Via Santa Sofia 64, 95123 Catania, Italy

**Keywords:** open quantum systems, synchronization, superconducting devices

## Abstract

A system consisting of two qubits and a resonator is considered in the presence of different sources of noise, bringing to light the possibility of making the two qubits evolve in a synchronized way. A direct qubit–qubit interaction turns out to be a crucial ingredient, as well as the dissipation processes involving the resonator. The detrimental role of the local dephasing of the qubits is also taken into account.

## 1. Introduction

Synchronization of physical systems plays an important role in many fields [1]. It consists of a dynamical alignment of systems, which means that different physical systems, characterized by different natural frequencies, due to some coupling, turn out to evolve in such a way that they exhibit a common frequency, usually different from the natural ones. The systems can be classical, as for example two clocks or metronomes [2,3], people walking on a bridge [4], or biological systems [5], but they can also be quantum. The typical situation involves two or more interacting quantum oscillators, possibly forming a network, where clusters of synchronized oscillators can be obtained [6,7,8]. In the realm of quantum mechanics, also two-level systems have been considered, as for example the case of two interacting two-state systems undergoing dissipation, which dynamically align after a certain time [9,10], or two atoms in a cavity coupled through the relevant mode [11]. In all such cases, the key ingredients to obtain synchronization are the formation of correlations (typically in the form of entanglement) and dissipation, which drives the system towards suitable superpositions of a limited number of states sharing one frequency or different frequencies, but very close ones. Indeed, generally speaking, the persistence of quantum correlations and entanglement in dissipating systems even at equilibrium has extensively been proven [12,13,14], but in out-of-equilibrium systems, such correlations can induce dynamical alignment. Recently, the possibility of obtaining the synchronization of two quantum harmonic oscillators coupled to the same dissipating qubits has been predicted [15]. Here, we want to analyze the complementary situation, consisting of two qubits interacting with a dissipating harmonic oscillator, which are driven to synchronization. In fact, in standard applications, for example in quantum information and technologies, qubits play a fundamental role. Therefore, realizing networks of synchronized qubits that exchange information, instead of the typical networks of oscillators, could be of significant usefulness.

There are several physical scenarios that one can focus on, but superconducting devices are one of the most promising. For example, over the last few decades, circuit QED involving superconducting artificial atoms has proven to be a more versatile scenario than the more traditional counterpart, i.e., cavity QED [16,17]. Superconducting circuits offer for example the possibility to reach a strong coupling regime where the joint system becomes anharmonic, allowing experiments in nonlinear optics and quantum information at the single-photon level [18]. Superconducting devices can be fabricated using modern integrated circuit technology, and their properties, as for example their energies, can be adjusted in situ and determined by circuit parameters, allowing for implementing devices with the desired features [17]. These systems thus offer a rich space of parameters and possible operation regimes allowing for the realization of a plethora of Hamiltonian models involving artificial atoms (including qubits as a special case) and resonators [19]. Moreover, superconducting quantum circuits are privileged candidates for large-scale quantum computing, and they have been used or proposed to implement quantum gates [20,21], as well as to generate entangled states [22], to show the violation of Bell-type inequalities [23], or to study thermodynamics at a quantum level [24,25,26,27].

Similar to natural atoms, superconducting artificial atoms are plagued by the presence of environments, so that generally, one has to take into account both dissipation and decoherence. In this case also, however, we can explore different regions of the parameters characterized by the nonunitary dynamics of the system. Indeed, different values and hierarchies of the relevant dissipation and dephasing rates can be considered, depending for example on whether we are considering transmonic qubits coupled to a waveguide [28], charge qubits coupled to a superconductor resonator [19], or other possible configurations [29,30,31]. The key tool to take into account environmental effects is a master equation. Generally speaking, in the case of different interacting physical systems, the master equation can be obtained with a phenomenological approach, by summing up the dissipators associated with different sources of noise that the single subsystems are subjected to or by deriving the microscopic master equation starting from the complete system–environment Hamiltonian, including the interaction between the subsystems from the beginning [32]. In superconducting devices, microscopic models for the dephasing mechanisms are available [33], which could be reconsidered in the presence of the interaction of the qubits with each other and with the resonator. Generally speaking, in the weak damping limit, the predictions coming from phenomenological and microscopic models essentially coincide [34,35]. Therefore, since in this paper, we will focus on the weak damping, for the sake of simplicity, we will use a phenomenological approach as in Blais et al. [19].

Dissipation is not necessarily a detrimental occurrence, since in some cases, it can even help to obtain interesting physical behaviors, as mentioned before in connection with dynamical alignment. In fact, we show in this paper that a dissipating resonator can drive two qubits interacting with it in a synchronized state of motion. The material is organized as follows. In Section 2, the Hamiltonian model describing the system, as well as the master equation governing its dynamics are presented. Section 3 is devoted to the discussion of the appearance of possible protected states, and under appropriate conditions, the occurrence of synchronization phenomena is predicted. Numerical simulations corroborate the theoretical analysis. The detrimental effect of local qubit dephasing and dissipation is also discussed. Then, in Section 4, the peculiarity of microcanonical evolutions under pure dephasing is discussed and analyzed. Finally, in Section 5, we give some conclusive remarks.

## 2. The Model

### 2.1. Hamiltonian and Dissipators

Our model consists of two qubits with different energy gaps interacting with a quantum harmonic oscillator (resonator) whose frequency is, in general, different from those of the two qubits. We also consider a possible direct interaction between the qubits:

(1) $$H=\sum_{j=A,B}\frac{\Omega_{j}}{2}\sigma_{z}^{j}+\Omega_{r}a^{†}a+\sum_{j=A,B}\kappa_{j}\left(\sigma_{-}^{j}a+\sigma_{-}^{j}a^{†}\right)+\kappa_{AB}\left(\sigma_{-}^{A}\sigma_{+}^{B}+\sigma_{+}^{A}\sigma_{-}^{B}\right)\;,$$

where $\Omega_{A}$ and $\Omega_{B}$ are the natural frequencies of the qubits described by the relevant Pauli operators $\sigma_{z}^{j}$, $\sigma_{x}^{j}$, and $\sigma_{y}^{j}$; $\Omega_{r}$ is the natural frequency of the oscillator described by the annihilation and creation operators *a* and $a^{†}$ and by the number operator $a^{†}a$; $\kappa_{A}$ and $\kappa_{B}$ are the qubit–resonator coupling strengths, while $\kappa_{AB}$ is the qubit–qubit coupling strength; both couplings involve the circular Pauli operators $\sigma_{\pm }^{j}=\left(\sigma_{x}\pm i\sigma_{y}\right)/2$. The qubit–resonator terms are given in the rotating wave approximation, which is a necessary approximation since it influences the structure of the eigenstates and, then, as we will see, the structure of the stable states, which are responsible for the synchronization processes. This approximation is valid because of the fact that, in the physical conditions we focus on, the qubit–resonator coupling constant is much smaller than the natural frequencies of the qubits and oscillator.

Since all the parts of the system interact with the environment, different sources of noise are present whose effects can be effectively described through a phenomenological master equation involving local dephasing and dissipation for the qubits and dissipation for the resonator [19]:

(2) $$\dot{\rho }=-i\left[H,\rho \right]+\gamma_{r}D\left[a\right]\rho +\sum_{j=A,B}\gamma_{j}D\left[\sigma_{-}^{j}\right]\rho +\sum_{j=A,B}{\tilde{\gamma }}_{j}D\left[\sigma_{z}^{j}\right]\rho \;,$$

where:

(3) $$D\left[X\right]\rho =X\rho X^{†}-\frac{1}{2}\left\{X^{†}X,\rho \right\}\;,$$

and $\gamma_{r}$, $\gamma_{A}$, and $\gamma_{B}$ are the decay rates associated with the dissipation processes of the resonator, qubit *A* and qubit *B*, respectively, while ${\tilde{\gamma }}_{j}$ (j=A,B) are the rates of the local dephasing processes of the two qubits. We observe that there is no thermal pumping (which would imply terms such as $D[a^{†}]$ and $D[\sigma_{+}^{j}]$), due to the fact that superconducting devices usually operate at a low temperature.

Generally speaking, one could wonder whether the presence of interactions between the parts of the system can change the form of the master equation, in the sense that, starting from a microscopic model of the interaction between the whole system and the environment and considering the interactions between the subsystems (each qubit and the resonator), one reaches the so-called microscopic master equation [32], which is proven to differ from the one obtained by evaluating the dissipator before considering the interaction between the subsystems. Nevertheless, since deviations between the two approaches occur in the high-decay regime [34,35], while we focus on the weak-damping limit, for the sake of simplicity, we use the master equation (2).

### 2.2. Conservation of the Excitation Number

An important property of the Hamiltonian in (1) is that the total number of excitations,

(4) $$\hat{N}=a^{†}a+\sigma_{z}^{A}+\sigma_{z}^{B}+2\;,$$

is a constant of motion, due to the fact that both the free terms and the interaction terms in the rotating wave approximation conserve it. Here, the constant 2 allows for having only positive eigenvalues.

This fact implies a block structure for the Hamiltonian involving quadruplets, when the number of excitations is greater than unity:

(5) $$|n\rangle |--\rangle \;,\;\;\;|n-1\rangle |+-\rangle \;,\;\;\;|n-1\rangle |-+\rangle \;,\;\;\;|n-2\rangle |++\rangle \;,\;n\geq 2\;.$$

Then, there is also the triplet with one-excitation states,

(6) $$|1\rangle |--\rangle \;,\;\;\;|0\rangle |+-\rangle \;,\;\;\;|0\rangle |-+\rangle \;,$$

and the singlet, given by the sole ground state $|G\rangle \equiv \;|0\rangle |--\rangle$, whose energy is $E_{G}=-\left(\Omega_{A}+\Omega_{B}\right)/2$.

In the presence of dephasing, the number operator is still conserved, since the operators $\sigma_{z}^{j}$ commute with $\hat{N}$. On the contrary, when dissipation is present, whether involving the qubits, the oscillator, or both, subspaces with different numbers of excitations are incoherently coupled and the number operator is not conserved.

## 3. Protected States and Qubit Synchronization

### 3.1. Theoretical Analysis

Let us focus on the case $\gamma_{A}=\gamma_{B}={\tilde{\gamma }}_{A}={\tilde{\gamma }}_{B}=0$ and $\gamma_{r}\neq 0$. Therefore, we have two two-state systems (the qubits) interacting with a dissipating oscillator (the resonator). Recently, the complementary situation consisting of two oscillators coupled with a dissipating two-state system has been analyzed [15], and it has been brought to light that, under suitable hypotheses, the two oscillators reach an almost stationary regime where they oscillate at the same frequency, thus leading to their synchronization. The reason for this occurrence is that there is the possibility to define two new modes (not necessarily the normal modes), one of which is coupled to the two-state system, then ‘indirectly’ undergoing dissipation, while the second one is decoupled and then insensitive to dissipation. After a long time, the dissipating mode loses all its energy, while the other one persists, imposing that the two oscillators move with a common frequency.

We try here to reproduce a similar behavior, driving the two qubits towards common oscillations corresponding to some protected transitions. Basically, we look for a decoherence-free subspace [36,37]. In order to be insensitive to the noise, a quantum state should belong to the kernel of the dissipator (which implies it does not have a direct coupling to the environment) and to the kernel of the qubit–oscillator coupling (in order to avoid indirect coupling to the environment), thus obtaining a state that is interaction-free [38,39] with respect to the qubit–resonator coupling. By imposing the second condition,

(7) $$\left[a\left(\kappa_{A}\sigma_{+}^{A}+\kappa_{B}\sigma_{+}^{B}\right)+a^{†}\left(\kappa_{A}\sigma_{-}^{A}+\kappa_{B}\sigma_{-}^{B}\right)\right]|P\rangle =0\;,$$

we find:

(8) $$|P\rangle =\cos\theta |0\rangle |+-\rangle +\sin\theta |0\rangle |-+\rangle \;,\;\tan\theta =-\kappa_{A}/\kappa_{B}\;,$$

as the only possible solution. This state (i.e., the corresponding projector) belongs to the kernel of the dissipator, since it factorizes the ground state $|0\rangle$. However, this is still not enough to prevent the waste of energy from the state $|P\rangle$. Indeed, if it is coupled through the other Hamiltonian terms (even free terms) to other states that are noise-sensitive, it can effectively decay. Therefore, we tune the strength of the qubit–qubit interaction to a specific value, which allows for $|P\rangle$ to be an eigenstate of the Hamiltonian. We in particular require that $H|P\rangle =E_{P}|P\rangle$, with $E_{P}$ to be determined. Since $H|P\rangle =\left[\left(-\kappa_{B}\left(\Omega_{A}-\Omega_{B}\right)/2+\kappa_{A}\kappa_{AB}\right)|0\rangle |+-\rangle +\left(\kappa_{A}\left(\Omega_{B}-\Omega_{A}\right)/2-\kappa_{B}\kappa_{AB}\right)|0\rangle |-+\rangle \right]/{\left(\kappa_{A}^{2}+\kappa_{B}^{2}\right)}^{1/2}$, we obtain:

(9) $$\kappa_{AB}=\frac{\left(\Omega_{A}-\Omega_{B}\right)\kappa_{A}\kappa_{B}}{\kappa_{A}^{2}-\kappa_{B}^{2}}\;,$$

(10) $$E_{P}=\frac{\left(\Omega_{B}-\Omega_{A}\right)\left(\kappa_{A}^{2}+\kappa_{B}^{2}\right)}{2(\kappa_{A}^{2}-\kappa_{B}^{2})}\;.$$

By tuning the qubit–qubit coupling constant $\kappa_{AB}$ to the value of (9), we obtain that the state $|P\rangle$ turns out to be protected from noise. There is another noise-insensitive state, which is the ground state $|G\rangle$. Therefore, after a long time, the only two surviving states are $|P\rangle$ and $|G\rangle$, and the system evolution is characterized by a single frequency, which is the $|P\rangle -|G\rangle$ transition frequency. It is interesting to note that under the condition $\kappa_{B}/\kappa_{A}=\sqrt{\Omega_{B}/\Omega_{A}}$, the energies $E_{P}$ and $E_{G}$ turn out to be equal, which implies the absence of oscillations in the long-time regime.

It is the case to observe that when $\kappa_{A}=\kappa_{B}$, the eigenvalue equation can be satisfied for any value of $\kappa_{AB}$, provided $\Omega_{A}=\Omega_{B}$. However, this is a trivial case that we are not interested in, since the two qubits would be synchronized from the beginning, having the same natural frequencies. Finally, we emphasize that there is no solution for $\kappa_{A}=\kappa_{B}$ and $\Omega_{A}\neq \Omega_{B}$.

### 3.2. Simulations

Our theoretical analysis is supported by numerical calculations. Since the master equation describing our system is time-independent, its numerical resolution can be easily performed through the evaluation of the exponential of the matrix representing the master equation, multiplied by the time *t*. Concerning the parameters, we considered typical values for the natural frequencies and the coupling constants [16,17,19]. The first ones should lie in the range 5–15 GHz, while the qubit–resonator coupling strength should lie in the range 10–200 MHz. Compatibly (on a $\kappa_{A}=100$ MHz basis), we mainly considered $\Omega_{A}/\kappa_{A}=55$, $\Omega_{B}/\kappa_{A}=70$, $\Omega_{r}/\kappa_{A}=64$, while $\kappa_{B}$ is of the same order of $\kappa_{A}$. Regarding the decay and dephasing rates, the local ones related to the qubits can be made rather small, having 0.02 MHz for local qubit dissipation and 0.3 MHz for local qubit dephasing, compatible with $\gamma_{j}/\kappa_{A}∼0.0002$ and ${\tilde{\gamma }}_{j}/\kappa_{A}∼0.003$. The resonator decay rate can be made even smaller than the qubit counterparts. Nevertheless, since we want to use the dissipation process of the resonator to induce qubit synchronization, we require the use of a less protected resonator (which is clearly always in the grasp of experimentalists) in order to have $\gamma_{r}$ larger than the other rates. In most simulations, we assumed $\gamma_{r}/\kappa_{A}=0.5$.

**Figure 1:**
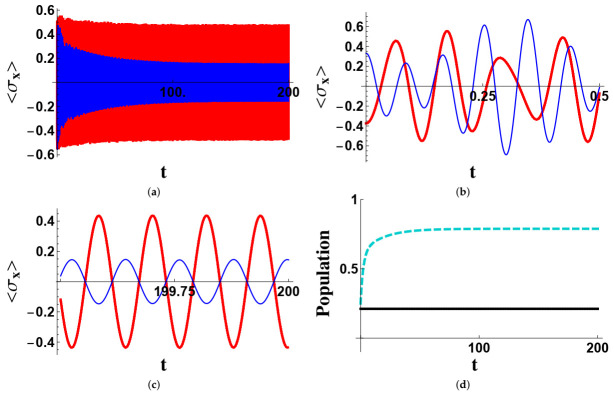
Time evolution of the mean values $\langle \sigma_{x}^{A}\rangle$ (bold red line) and $\langle \sigma_{x}^{B}\rangle$ (thin blue line) in different time windows and of the populations of the states $|P\rangle$ (solid black line) and $|G\rangle$ (cyan dashed line) in the wide range. Time is given in units of $\kappa_{A}^{-1}$. The initial state of the system is $|\psi (0)\rangle =\left(|P\rangle +|G\rangle \right)/2+|1\rangle |--\rangle /\sqrt{2}$. The parameters are (in units of $\kappa_{A}$): $\Omega_{A}=55$, $\Omega_{B}=70$, $\Omega_{r}=64$, $\kappa_{B}=3$, $\gamma_{r}=0.5$, $\gamma_{A}=\gamma_{B}={\tilde{\gamma }}_{A}={\tilde{\gamma }}_{B}=0$. The coupling $\kappa_{AB}$ is determined by the decoupling condition in (9). The three time-windows considered for the expectation value of $\langle \sigma_{x}^{j}\rangle$ are the wide-rage (**a**), the short-time (**b**) and the long-time (**c**). The populations of the protected and ground states are considered only in the wide-range (**d**).

**Figure 2:**
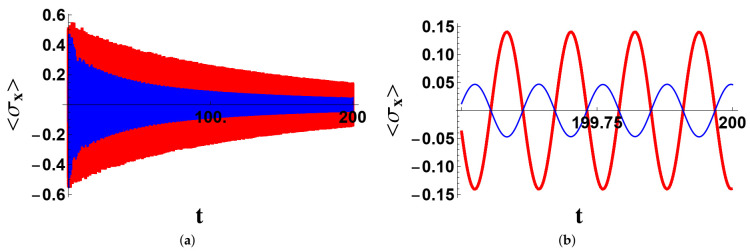
The same initial condition and parameters of Figure 1, except for the local qubit dephasing and dissipation rates: $\gamma_{A}=\gamma_{B}=0.0002$ and ${\tilde{\gamma }}_{A}={\tilde{\gamma }}_{B}=0.003$. Only the wide-range (**a**) and long-time (**b**) plots are reported.

In Figure 1, we report the appearance of a synchronized evolution of the two qubits, singled out by the time evolution of the two expectation values $\langle {\hat{\sigma }}_{x}^{j}\rangle$, j=A,B, and explained in terms of the populations of the two stationary states $|P\rangle$ and $|G\rangle$. The system was assumed to be prepared in the state $|\psi (0)\rangle =\left(|P\rangle +|G\rangle +\sqrt{2}|1\rangle |--\rangle \right)/2$, which is an equal-weight superposition of the two protected states ‘soiled’ by a nonprotected one. We assumed no local qubit dissipation or dephasing. The wide-range plot (Figure 1a) shows a general loss of energy of the system associated with a diminishing of the amplitude of ${\hat{\sigma }}_{x}^{B}$. The short-time plot (Figure 1) shows that the two signals are very different in the very first part of the evolution, while after a long time, as shown in Figure 1c, the two expectation values become two clean sinusoidal signals with the same frequency. From Figure 1d, we inferred that the dynamical alignment of the two qubits is concomitant with the increase of the population of the ground state, to the point where only $|P\rangle$ and $|G\rangle$ are present in the state of the system. In Figure 2, we consider the effects of the local dissipation and dephasing of the qubits. It is well visible that the amplitudes of the oscillations are smaller than in the previous case, but not dramatically smaller, making the effect still appreciable.

**Figure 3:**
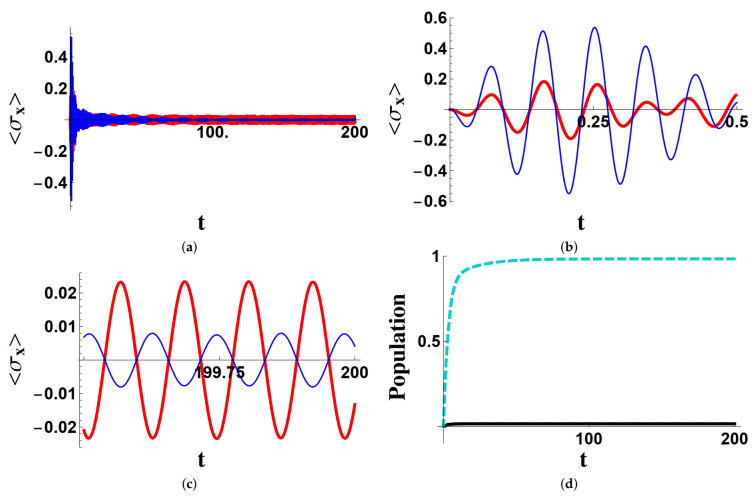
Time evolution of the mean values $\langle \sigma_{x}^{A}\rangle$ (bold red line) and $\langle \sigma_{x}^{B}\rangle$ (thin blue line) in different time windows and of the populations of the states $|P\rangle$ (solid black line) and $|G\rangle$ (cyan dashed line) in the wide range. Time is given in units of $\kappa_{A}^{-1}$. The initial state of the system is $|\psi (0)\rangle =\left(|2\rangle |--\rangle +|1\rangle |--\rangle \right)/\sqrt{2}$. The parameters are (in units of $\kappa_{A}$): $\Omega_{A}=55$, $\Omega_{B}=70$, $\Omega_{r}=64$, $\kappa_{B}=3$, $\gamma_{r}=0.5$, $\gamma_{A}=\gamma_{B}={\tilde{\gamma }}_{A}={\tilde{\gamma }}_{B}=0$. The coupling $\kappa_{AB}$ is determined by the decoupling condition in (9). The three time-windows considered for the expectation value of $\langle \sigma_{x}^{j}\rangle$ are the wide-rage (**a**), the short-time (**b**) and the long-time (**c**). The populations of the protected and ground states are considered only in the wide-range (**d**).

In Figure 3, we analyze a situation similar to that of Figure 1, but in connection with a different initial condition: $|\psi (0)\rangle =\left(|2\rangle |--\rangle +|1\rangle |--\rangle \right)/\sqrt{2}$. Since the amount of coherence $\langle P|\rho \left(t\right)|G\rangle$ (with ρ(t), the state of the system at time *t*) at t=0 is very small, its final value turns out to be small even at a long time, which implies oscillations with very small amplitudes. It is also well visible that the final population of the state $|P\rangle$ is rather small, though not vanishing.

**Figure 4:**
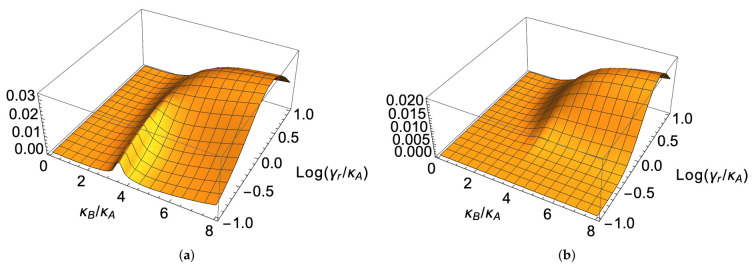
Modulus of the coherence $\langle P|\rho \left(t\right)|G\rangle$ at time $t=200/\gamma_{r}$ as a function of $\kappa_{B}/\kappa_{A}$ and $\log(\gamma_{r}/\kappa_{A})$, for the initial state $|\alpha \rangle |--\rangle$, with α=1, in the absence of dephasing (**a**) and in the presence of dephasing (**b**). The relevant parameters are (in units of $\kappa_{A}$): $\Omega_{A}=55$, $\Omega_{B}=70$, $\Omega_{r}=64$, $\gamma_{A}=\gamma_{B}=0$, and $\kappa_{AB}$ is determined by the decoupling condition in (9). In (**a**), we have $\gamma_{A}=\gamma_{B}={\tilde{\gamma }}_{A}={\tilde{\gamma }}_{B}=0$, while in (**b**), $\gamma_{A}=\gamma_{B}=0.0002$ and ${\tilde{\gamma }}_{A}={\tilde{\gamma }}_{B}=0.003$.

In Figure 4, we plot the long-time coherence as a function of $\kappa_{B}$ and $\gamma_{r}$ (here forcing the model to the strong-damping limit, since we reach the value $\gamma_{r}=10\kappa_{A}$), for two different initial conditions. In Figure 4a, a coherent state is considered for the resonator, while the two qubits are in their natural ground state: $|\psi (0)\rangle =\;|\alpha \rangle |--\rangle$, with α=1; in Figure 4b, the same situation is reported, but in the presence of local qubit dissipation and dephasing.

## 4. Dephasing

The appearance of the synchronized motion of the two qubits is based on the interaction between the two-state systems and the dissipating resonator, which drives most of the two-qubit states towards the ground state, leaving only a specific two-state superposition involving only a transition frequency. Local qubit dissipation and dephasing processes instead tend to destroy synchronization, as is well visible, for example, in Figure 2 and Figure 4.

Pure dephasing itself is an interesting phenomenon from the thermodynamical point of view, since it is the main mechanism of the relaxation of a system into a microcanonical state. Usually, dephasing does not alter the energy of the system and induce a random distribution of the states with equal energies. Since, in our case, the dephasing mechanism does not conserve the energy of the system, but only its total number of excitations, it is interesting to observe the structure of the equilibrium state in such a situation. Let us then focus on the pure dephasing (thus, assuming $\gamma_{r}=\gamma_{A}=\gamma_{B}=0$):

(11) $$\dot{\rho }=-i\left[H,\rho \right]+\sum_{j}{\tilde{\gamma }}_{j}\left(\sigma_{z}^{j}\rho \sigma_{z}^{j}-\rho \right).$$

The microcanonical state related to a subspace with a fixed number of excitations is a stationary state: $\rho =g_{n}^{-1}{\hat{\Pi }}_{n}$ ⇒ $-i\left[H,\rho \right]+\sum_{j}{\tilde{\gamma }}_{j}\left(\sigma_{z}^{j}\rho \sigma_{z}^{j}-\rho \right)=0$, where ${\hat{\Pi }}_{n}$ denotes the projector onto the subspace with *n* excitations and $g_{n}$ is the relevant degeneracy.

It is possible to prove that this is the only possible stationary state, unless specific conditions (given below) are satisfied. Indeed, assume $\rho_{n}=\sum_{k}p_{nk}|\psi_{nk}\rangle \langle \psi_{nk}|$, where $\sum_{k}p_{nk}=1$ and $|\psi_{nk}\rangle$’s form an orthonormal basis of the subspace with *n* excitations. The action of the Lindbladian gives:

(12) $$\begin{array}{ccc} & - & i(\sum_{k}p_{nk}|\psi_{nk}\rangle \langle \phi_{nk}|-\sum_{k}p_{nk}|\phi_{nk}\rangle \langle \psi_{nk}|)\\ & + & \sum_{j}{\tilde{\gamma }}_{j}\left[\sum_{k}p_{nk}\sigma_{z}^{j}|\psi_{nk}\rangle \langle \psi_{nk}|\sigma_{z}^{j}-\sum_{k}p_{nk}|\psi_{nk}\rangle \langle \psi_{nk}|\right]\;, \end{array}$$

where $|\phi_{nk}\rangle =H|\psi_{nk}\rangle$ are non-normalized states. In order to make this quantity vanish, the first two sums must give zero, which implies $|\phi_{nk}\rangle \propto \;|\psi_{nk}\rangle$, i.e., the states $|\psi_{nk}\rangle$ are Hamiltonian eigenstates. In order to obtain zero, the other two terms should compensate each other. Since the states $|\psi_{nk}\rangle$ are orthogonal, the only possibility is that, for every *k*, $\sigma_{z}^{j}|\psi_{nk}\rangle =e^{i\varphi_{nkj}}|\psi_{nm_{j}}\rangle$, provided $p_{nk}=p_{nm}$. Of course, in such a case, one also has $\sigma_{z}^{j}|\psi_{nm}\rangle =e^{-i\varphi_{nkj}}|\psi_{nk_{j}}\rangle$. Therefore, unless specific eigenstates of the Hamiltonian exist that are eigenstates of both the $\sigma_{z}^{j}$ or are mapped by such operators to other Hamiltonian eigenstates, there is no stationary state different from the microcanonical one.

**Figure 5:**
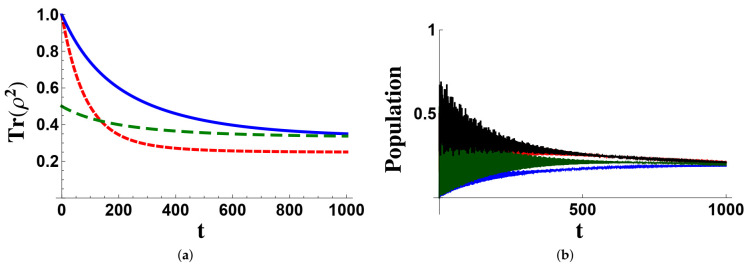
In (**a**), we report the time evolution of the linear entropy for different initial states: $|1\rangle |--\rangle$ (blue solid line), $|2\rangle |--\rangle$ (red dotted line), and the mixed state $(|0\rangle \langle 0|⊗|-+\rangle \langle -+|+|0\rangle \langle 0|⊗|+-\rangle \langle +-|)/2$ (green dashed line). In (**b**), the time evolutions of the populations of the states $|2\rangle |--\rangle$ (red line), $|1\rangle |+-\rangle$ (blue line), $|1\rangle |-+\rangle$ (green line), and $|0\rangle |++\rangle$ (black line) are plotted, when the system is prepared in $|\psi (0)\rangle =\left(|0\rangle |++\rangle +|2\rangle |--\rangle \right)/\sqrt{2}$. Time is given in units of $\kappa_{A}^{-1}$. The coupling $\kappa_{AB}$ is determined by the decoupling condition, while the other parameters are (in units of $\kappa_{A}$): $\Omega_{A}=55$, $\Omega_{B}=70$, $\Omega_{r}=64$, $\kappa_{B}=3$, ${\tilde{\gamma }}_{A}={\tilde{\gamma }}_{B}=0.003$, $\gamma_{r}=\gamma_{A}=\gamma_{B}=0$.

On the basis of the previous results, one can expect that when the system is prepared as an eigenstate of the number operator $\hat{N}$ (i.e., every state belongs to a multiplet with fixed *n*), the evolution naturally brings the system toward the microcanonical state, which is characterized by a maximum von Neumann entropy (S(ρ)≡−Tr(ρlogρ)→$S\left(g_{n}^{-1}{\hat{\Pi }}_{n}\right)=\log g_{n}$) and a minimum linear entropy ($P\left(\rho \right)\equiv \mathrm{Tr}\rho^{2}$→$P\left(g_{n}^{-1}{\hat{\Pi }}_{n}\right)=g_{n}^{-1}$). In Figure 5a, we show the time behavior of the linear entropy when the system is prepared in different states. The two initial conditions belonging to the triplet with n=1 lead to an asymptotic purity of 1/3, while the initial state belonging to the quadruplet with n=2 evolves in such a way to give a purity equal to 1/4, as expected. In Figure 5b, an example of the evolution of the populations of states belonging to the quadruplet with n=2 is given, assuming that the system is prepared in a state with two excitations. As is well visible, all populations eventually reach the value 1/4.

The peculiarity of the microcanonical state the system approaches is that it does not describe a situation where all the eigenstates of the Hamiltonian with the same energy have equal probability. Since the given constraint relates to the number of excitations, the uniform probability distribution refers to the states with the same number of excitations.

## 5. Discussion and Conclusions

In this paper, we studied a pseudo-Dicke model describing the interaction between a harmonic oscillator (the resonator) and two two-state systems (qubits), which can be realized with superconducting devices. The qubit–resonator coupling was assumed to be in the rotating wave approximation, which allows for the conservation of the total number of excitations. Besides this interaction, a direct qubit–qubit coupling was considered. Moreover, since all the components were subjected to an interaction with the environment, dephasing and dissipation processes were also included in the model.

We see that it is possible to identify a state that is insensitive to the qubit–resonator interaction and that, by suitably tuning the qubit–qubit coupling, such a state can be made an eigenstate of the Hamiltonian, hence allowing for the relevant qubit state to be stationary. This state and the ground state of the system form a protected subspace. Therefore, after a large enough time, the system inevitably relaxes toward a mixture involving these two states with a possible residual coherence. As a consequence, the two-qubit system exhibits oscillations at the frequency that separates the protected and the ground states. This leads to an effective synchronization, which provides the complementary scenario to that analyzed in [15], where two oscillators synchronized due to the common interaction with a dissipating two-state system. The direct coupling between the qubits plays a crucial role, since it allows for avoiding transitions from the state that is insensitive to the qubit–resonator coupling towards states that are affected by that interaction term.

We also analyzed the role of local qubit dissipation and dephasing processes, showing that they are able to damage the processes inducing synchronization. On the other hand, dephasing itself makes the system relax towards a pseudo-microcanonical state, which is characterized by the equiprobability of the states with the same number of excitations. This analysis shows how rich the superconducting-device dynamics can be.

## Data Availability

Data sharing not applicable.
